# Metabolic profiling reveals the heterogeneity of vascular endothelial function phenotypes in individuals at extreme cardiovascular risk†

**DOI:** 10.1039/c9ra05526f

**Published:** 2019-09-23

**Authors:** Baoyu Mao, Yanshan Yi, Qiuyan Mo, Chunxiu Yang, Qiuan Zhong

**Affiliations:** Guangxi Colleges and Universities Key Laboratory of Prevention and Control of Highly Prevalent Diseases, Guangxi Medical University School of Public Health 22 Shuangyong Road Nanning 530021 China qazhong@gxmu.edu.cn; Department of Epidemiology, Guangxi Medical University School of Public Health 22 Shuangyong Road Nanning 530021 China

## Abstract

Maladapted vascular endothelial metabolism in the context of endothelial function differing in phenotype remains unknown, which limits our understanding of the heterogeneous pathogenesis of atherosclerotic cardiovascular disease (CVD). This study aimed to profile serum metabolic alterations of different vascular endothelial function phenotypes in asymptomatic adults at extreme cardiovascular risk. In addition to 12 CVD patients, 103 individuals free of CVD were categorized as having normal endothelial function (NEF) (*n* = 30), cardiovascular risk-promoting endothelial function (PEF) (*n* = 18), cardiovascular risk-resistant endothelial function (REF) (*n* = 25), and vulnerable endothelial function (VEF) (*n* = 30). Serum metabolic profiles were detected using gas chromatography time-of-flight/mass spectrometry and multivariate statistics. Compared to the NEF group, a total of 17, 17, 22, and 13 differential metabolites were identified in the PEF, REF, VEF, and CVD groups, respectively. Of the altered metabolic pathways, multiple pathways were consistent between the PEF and CVD groups, including pyrimidine metabolism, starch and sucrose metabolism, aminoacyl-tRNA biosynthesis, arginine and proline metabolism, and d-glutamine and d-glutamate metabolism. Notably, a relative increase in low-calorie sugar in galactose metabolism was exclusively found in the REF group, and a relative increase in the ratio of acetyl-CoA to CoA was suggested in the VEF group based on elevated butanoate metabolism and reduced pantothenate and CoA biosynthesis. Our findings clearly indicate distinct metabolic patterns across groups with heterogeneous vascular endothelial function in the context of extreme cardiovascular risk, and improve our understanding of the pathogenic heterogeneity of early CVD in asymptomatic populations.

## Abstract 2

This metabolomics analysis has revealed the maladapted vascular endothelial metabolism across individuals with heterogeneous vascular endothelial function in the context of extreme cardiovascular risk.

## Graphical abstract

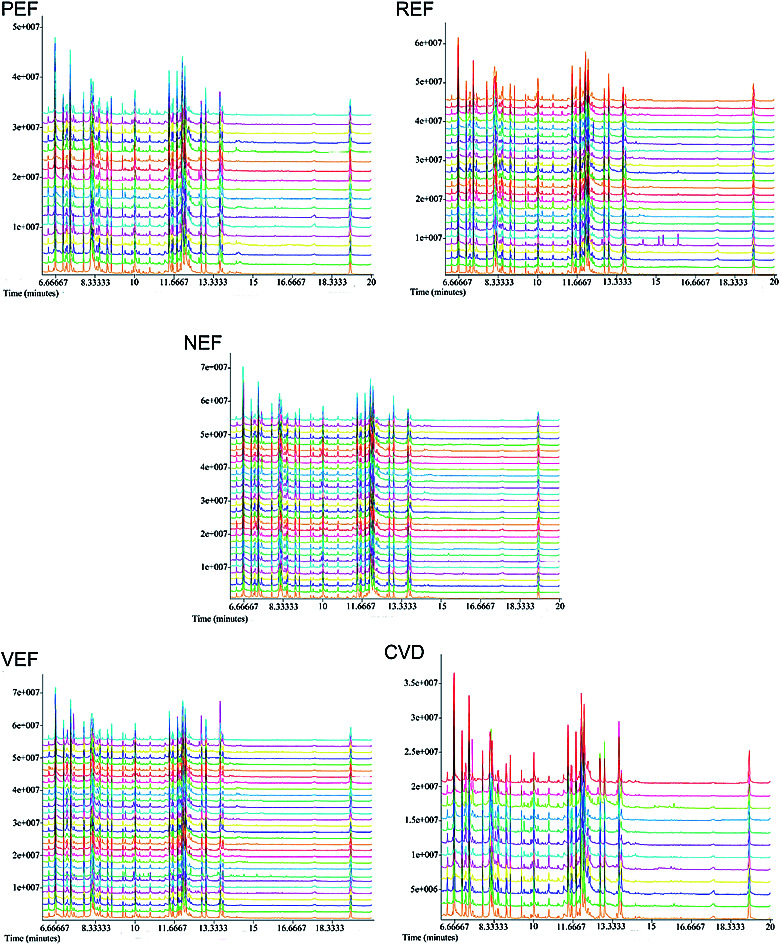

## Introduction

More attention is paid to populations at extreme cardiovascular risk. In fact, approximately 35% of individuals at high cardiovascular risk are less likely to develop atherosclerotic cardiovascular disease (CVD) events, while the risk of CVD events is elevated from 12% to 21% in individuals at low cardiovascular risk,^1,2^ suggesting a heterogeneous pathogenesis in atherosclerotic processes. Previous studies have also reported outlier individuals who did not have the expected number of CVD events according to their specific cardiovascular risk level;^3–6^ however, the underlying mechanisms are largely unclear. Indeed, as an outcome of a multistage, complex and chronic pathological process, different CVD types with notable pathophysiological differences exist.^7^ Thus far, studies have remained focused on the clinical stage of CVD, which may lead to an incomplete understanding of the heterogeneity in atherogenesis.

Vascular endothelial dysfunction, characterized mainly by the loss of nitric oxide (NO) bioavailability, is an initial manifestation in the pathological development of atherosclerotic CVD.^8–10^ More importantly, dysfunctional endothelium serves as a pathological indicator in diseased vasculature through metabolic signals.^11^ Therefore, knowledge of the endogenous metabolic profiles related to endothelial function under different risk profiles may be crucial to understanding the heterogeneity of atherogenesis. Increasing cardiovascular metabolomics evidence has recently emerged from patients with carotid atherosclerotic plaques and coronary artery diseases.^12–15^ However, metabolic profiling of early cardiovascular pathology, *i.e.*, vascular endothelial dysfunction, is still poorly understood in the asymptomatic population.

In this study, we aim to profile metabolic changes relevant to the heterogeneous vascular endothelial functions in the serum of asymptomatic individuals at extreme cardiovascular risk using an untargeted metabolomics approach based on gas chromatography time-of-flight/mass spectrometry (GC-TOF/MS) with high resolution and detection sensitivity.^16,17^ Additionally, the present analyses seek to explore the potential mechanism pathways involved in heterogeneous atherogenesis from metabolic clues.

## Methods

### Study population

A total of 899 subjects aged 20 to 82 years including 865 asymptomatic individuals and 34 CVD patients identified by written medical records were recruited from Rongan and Rongshui of Guangxi, China in 2015. Data were generated by a cardiovascular health survey including an interview questionnaire (age, sex, ethnicity, cigarette smoking, history of CVD and medication use), physical examination (brachial artery flow-mediated dilation (FMD), body mass index (BMI), heart rate and blood pressure) and fasting serum collection (metabolites, total cholesterol, triglycerides, low-density lipoprotein (LDL) cholesterol, high-density lipoprotein (HDL) cholesterol, glucose and C-reactive protein (CRP)). The health survey and all experiments with blood samples in this study were performed in strict accordance with the Declaration of Helsinki Principles. All participants provided informed consent, and the study protocols were approved by the Medical Ethics Committee of Guangxi Medical University.

### Cardiovascular risk estimation

The cardiovascular risk for asymptomatic participants aged 30–74 years was estimated using the general formula of the sex-specific 10 year Framingham risk score (FRS), which includes age, total cholesterol, HDL cholesterol, treated or untreated systolic blood pressure, current smoking and diabetes.^18^ The estimated cardiovascular risk was categorized as low (FRS ≤ 6%), moderate (FRS > 6% and ≤ 20%), or high (FRS > 20%).

### Vascular endothelial function assessment

Vascular endothelial function was assessed by brachial artery FMD. All participants abstained from using vasoactive medications, smoking, alcohol use, caffeine consumption, or eating a high-fat diet the day before the measurement. After resting for at least 15 min, measurements were performed using a UNEX EF38G high-resolution ultrasound system (UNEX Corporation, Nagoya, Japan) in a quiet room at a comfortable temperature. Details of the FMD measurement have been described elsewhere.^19^ The brachial FMD is expressed as the percent increase in maximum diameter after reactive hyperemia relative to the baseline brachial artery diameter. Endothelial function was categorized as good (FMD ≥ 10%), moderate (FMD ≥ 6% and <10%), or dysfunctional (FMD < 6%).

### Definition of vascular endothelial phenotypes

Four endothelial phenotypes were set up in asymptomatic participants. Normal endothelial function (NEF) was defined as having low cardiovascular risk and good endothelial function, cardiovascular risk-promoting endothelial function (PEF) was defined as having high cardiovascular risk and dysfunctional endothelial function, cardiovascular risk-resistant endothelial function (REF) was defined as having high cardiovascular risk but good endothelial function, and vulnerable endothelial function (VEF) was defined as having low cardiovascular risk but dysfunctional endothelial function.

For the metabolomics analysis, 30 individuals with NEF, 18 individuals with PEF, 25 individuals with REF, and 30 individuals with VEF were sampled with overall matching for age and sex from 680 candidates. Additionally, 12 patients with coronary artery disease were included for comparison of metabolites, in order to comprehensively assess the distinct metabolic patterns of endothelial function (ESI Fig. 1†). All subjects enrolled in the metabolomics analysis were free of any agent use for at least 3 days prior to the survey.

### Sample preparation for GC-TOF/MS

A 100 μL aliquot of serum was transferred into a 1.5 mL centrifuge tube and vortexed for 10 s after adding 0.35 mL of methanol and 20 μL of l-2-chlorophenylalanine as an internal standard. The mixture was centrifuged at 13 000 rpm for 15 min at 4 °C. Then, 0.4 mL of supernatant was transferred into a 2 mL silylated glass vial and dried in a vacuum concentrator without heating. A 50 μL aliquot of methoxy amination hydrochloride (20 mg mL^−1^ in pyridine) was subsequently added to the dried extracts, followed by incubation in an oven for 30 min at 80 °C after mixing gently. Then, 70 μL of bistrifluoroacetamide (containing 1% TCMS, v/v) was added to each mixture and incubated at 70 °C for 2 h. Finally, the mixture was cooled to room temperature, followed by the addition of 10 μL of FAMEs (standard mixture of fatty acid methyl esters, C8–C16: 1 mg mL^−1^; C18–C24: 0.5 mg mL^−1^ in chloroform) for GC-TOF/MS analysis.

### GC-TOF/MS analysis and data processing

GC-TOF/MS was performed using an Agilent 7890 gas chromatograph system (Agilent, USA) coupled with a Pegasus HT time-of-flight mass spectrometer (LECO, Saint Joseph, MI, USA). The system utilized a DB-5MS capillary column coated with 5% diphenyl cross-linked with 95% dimethylpolysiloxane (30 m × 250 μm inner diameter, 0.25 μm film thickness; J&W Scientific, Folsom, CA, USA). A 1 μL aliquot was injected in splitless mode, using helium as the carrier gas, with a 3 mL min^−1^ front inlet purge flow and a 1 mL min^−1^ gas flow rate through the column. The initial temperature of the column was kept at 50 °C for 1 min; it was then raised to 310 °C at a rate of 20 °C min^−1^ and maintained for 6 min. The temperatures of injection, the transfer line and the ion source were 280, 270 and 220 °C, respectively. The energy was set at 70 eV in electron impact mode. The mass spectrometry data were acquired at a rate of 20 spectra per second after a solvent delay of 366 s with a mass-to-charge (*m*/*z*) range of 50–500 in full-scan mode.

Raw data were obtained using Chroma TOF 4.3X software (LECO, Saint Joseph, MI, USA) and the LECO-Fiehn Rtx5 database after raw peak extraction, baseline data filtering and calibration, peak alignment, deconvolution analysis, peak identification, and peak area integration. The raw peaks were retained after further filtering by the interquartile range denoising method. Then, for missing values in the raw data, a simulation method with half of the minimum value was used to fill up the dataset. Finally, the remaining data were processed using an internal standard normalization method. The LECO/Fiehn Metabolomics Library was used to evaluate the accuracy of compound identification *via* a similarity value. The identified metabolites were further verified by searching the PubChem Compound database (http://pubchem.ncbi.nlm.nih.gov), the Kyoto Encyclopedia of Genes and Genomes (KEGG) database (http://www.genome.jp/kegg), and the Human Metabolome database (http://www.hmdb.ca).

### Statistical analysis

In total, 4 comparisons, including PEF *versus* NEF, REF *versus* NEF, VEF *versus* NEF and CVD *versus* NEF, were performed in this study. Briefly, after importing the metabolic data, including peak number (RT and *m*/*z* pairs), sample name (observation) and normalized peak area (variable), the SIMCA 14.1 software package (Umetrics, Umea, Sweden) was used for principal component analysis (PCA) and orthogonal projections to latent structures discriminant analysis (OPLS-DA). The PCA was used to characterize the overall distribution of the data matrix for all groups. A supervised OPLS-DA was used to discriminate the different metabolites between the comparison groups. The models were evaluated using 7-fold cross validation and permutation tests.

The metabolites that differed between comparison groups were selected according to the variable importance in the projection (VIP) value (VIP value > 1.0) and Student's *t*-test (*P* value < 0.05). The relevant metabolic pathways of the differential metabolites were searched *via* databases including the KEGG and the NIST (http://www.nist.gov/index.html). The differential metabolites were further exported to the online MetaboAnalyst (http://www.metaboanalyst.ca) for pathway analysis that integrated enrichment analysis and pathway topology analysis. Moreover, receiver operating characteristic (ROC) analysis was performed using STATA version 13.1 (StataCorp LP, College Station, TX, USA) to evaluate the accuracy of the discriminatory ability of differential metabolites.

## Results

### Characteristics of the study participants

The average FMD of the participants was 12.8% for NEF, 4.4% for PEF, 11.8% for REF, 4.0% for VEF, and 7.8% for CVD. Generally, individuals with PEF were more likely to have higher percentages or levels of current smoking, BMI, systolic blood pressure, and serum triglyceride, but lower levels of serum HDL cholesterol. Individuals with REF were more likely to have higher heart rates, serum total cholesterol, and serum LDL cholesterol. Individuals with VEF were more likely to have lower percentages or levels of current smoking, BMI, systolic blood pressure, heart rates, serum CRP, serum total cholesterol, serum triglyceride, serum LDL cholesterol, and diabetes but a higher level of serum HDL cholesterol. CVD patients were more likely to be older, have higher serum CRP, and have diabetes (ESI Table 1†).

### Compound profiles by GC-TOF/MS

**Fig. 1:**
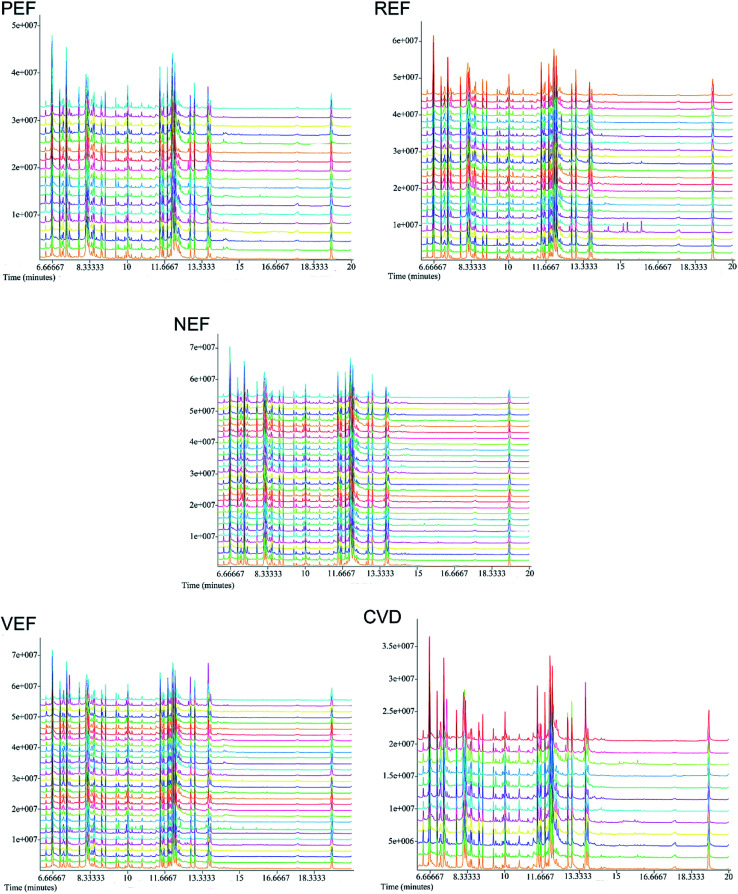
Representative GC-TOF/MS total ion chromatograms of the samples from the NEF, PEF, REF, VEF and CVD groups. GC-TOF/MS, gas chromatography time-of-flight/mass spectrometry; NEF, normal endothelial function; PEF, cardiovascular risk-promoting endothelial function; REF, cardiovascular risk-resistant endothelial function; VEF, vulnerable endothelial function; CVD, cardiovascular disease.

**Fig. 2:**
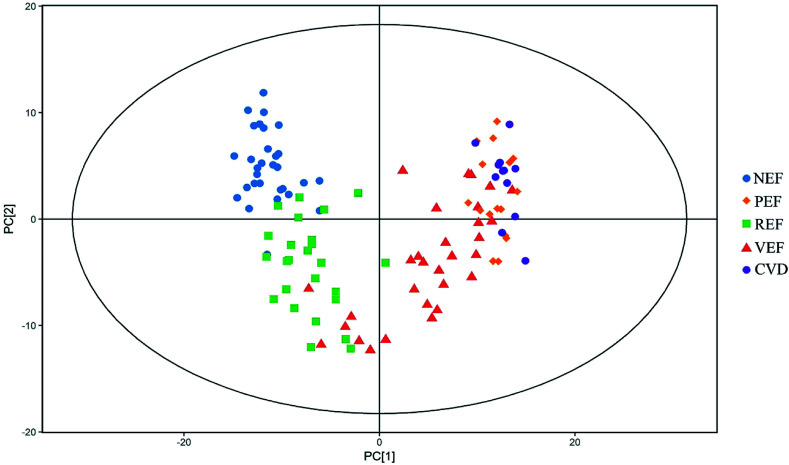
The PCA score plots of different endothelial function groups and the CVD group. PCA, principal components analysis; NEF, normal endothelial function; PEF, cardiovascular risk-promoting endothelial function; REF, cardiovascular risk-resistant endothelial function; VEF, vulnerable endothelial function; CVD, cardiovascular disease.

The typical total ion chromatograms (TICs) of the serum samples from the five groups are shown in Fig. 1. A total of 126 compounds were ultimately identified according to the LECO/Fiehn Metabolomics Library, the majority of which were endogenous metabolites including amino acids, organic acids, fatty acids, carbohydrates and nucleosides. Based on the PCA score plot, all samples were within the 95% Hotelling's *T*-squared ellipse, generally indicating separable clusters in the metabolic profiles of the five groups, except for between PEF and CVD (Fig. 2).

### Differential metabolites for different endothelial phenotypes

**Fig. 3:**
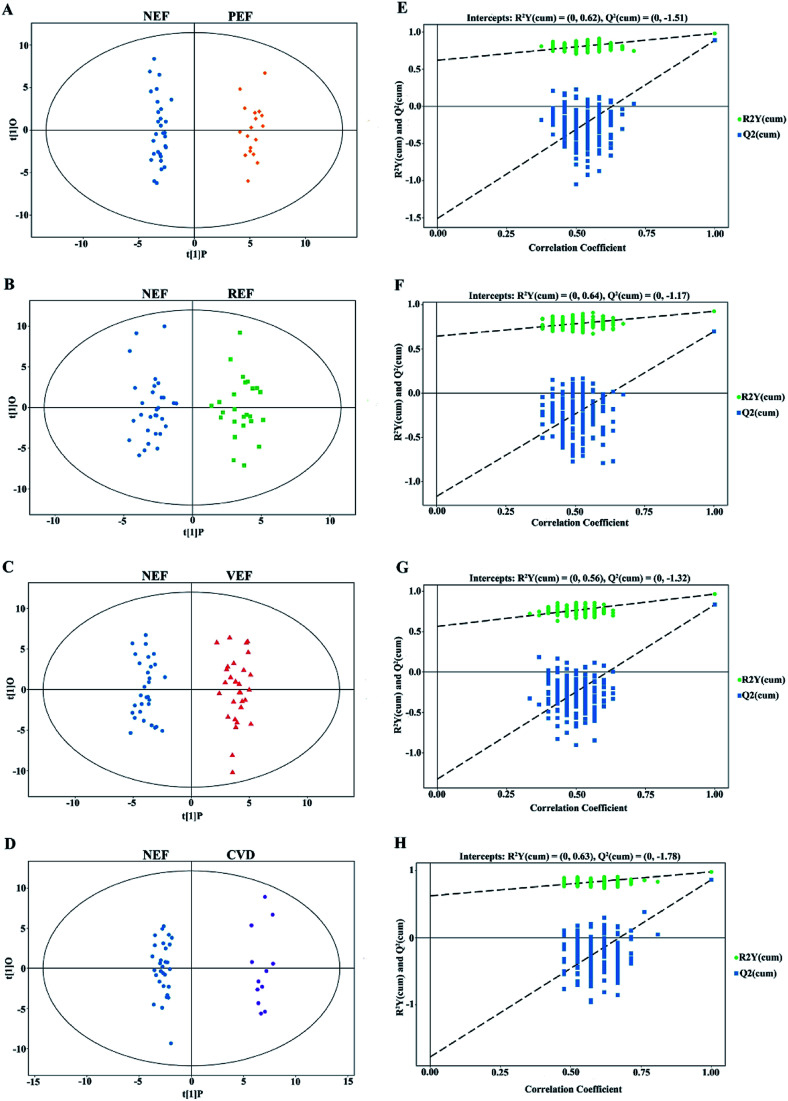
The OPLS-DA score plots comparing (A) PEF *versus* NEF, (B) REF *versus* NEF, (C) VEF *versus* NEF and (D) CVD *versus* NEF. The corresponding 7-fold cross validations are shown in (E–H), respectively. OPLS-DA, orthogonal projection to latent structures-discriminant analysis; NEF, normal endothelial function; PEF, cardiovascular risk-promoting endothelial function; REF, cardiovascular risk-resistant endothelial function; VEF, vulnerable endothelial function; CVD, cardiovascular disease.

In this study, clear separations are shown in all comparisons by the OPLS-DA models (Fig. 3A–D), with good fitness and prediction indicated by *R*^2^*Y* values ranging from 0.925 to 0.983 and *Q*^2^ values ranging from 0.697 to 0.892 (ESI Table 2†). For all comparisons, the permutation tests with low values of *R*^2^ and *Q*^2^ indicated robustness and a low risk of overfitting for the OPLS-DA models (Fig. 3E–H).

Altogether, 42 unique differential metabolites with VIP values > 1.0 and *P* values < 0.05 are shown in Table 1. Compared to NEF, a total of 17, 17, 22, and 13 metabolites were significantly altered in PEF, REF, VEF, and CVD, respectively. Among the differential metabolites, there were 6 and 11 metabolites for PEF, 7 and 10 metabolites for REF, 9 and 13 metabolites for VEF, and 1 and 12 metabolites for CVD that increased (fold change value >1) and decreased (fold change value <1), respectively. Moreover, 7, 6, 8, and 3 exclusive metabolites were found for PEF, REF, VEF, and CVD, respectively.

**Table 1:** Significantly different serum metabolites by comparison groups^a^

Metabolites	Similarity	R.T. (minutes)	VIP	*P* value	Fold change	Exclusive
**PEF *versus* NEF**						
Tryptophan	890	13.66	3.66	<0.001	2.51 × 10^−9^	
Urea	793	8.19	3.25	<0.001	6.15	
Monoolein	778	15.93	1.03	0.023	2.21	Yes
1-Monopalmitin	775	15.12	1.02	0.033	2.11	Yes
Uric acid	745	13.00	3.51	<0.001	8.96 × 10^−9^	
Sucrose	656	15.30	3.52	<0.001	6.44 × 10^−7^	
3-Aminoisobutyric acid	539	9.60	1.28	0.015	0.73	
Oxamide	475	8.77	1.20	0.045	1.28	Yes
Abietic acid	467	14.37	3.42	<0.001	1.50 × 10^−7^	
3-Hydroxynorvaline	466	9.02	1.17	0.046	0.88	Yes
Thymidine	455	10.65	2.41	<0.001	0.08	
2-Ketovaleric acid	403	7.60	1.23	0.018	1.27	Yes
Phthalic acid	393	11.02	1.03	0.010	1.80	Yes
Glutamic acid	315	10.59	1.03	<0.001	0.40	
Fructose	299	11.95	2.44	<0.001	0.07	
4-Methylcatechol	269	9.19	1.89	0.042	0.56	Yes
Acetol	263	11.13	1.12	0.008	0.73	

**REF *versus* NEF**						
Tagatose	938	11.85	1.38	<0.001	5.81	Yes
Arachidonic acid	850	14.30	1.70	0.015	1.27	Yes
Arachidic acid	681	14.55	2.62	0.007	0.41	
Sucrose	656	15.30	4.08	<0.001	6.30 × 10^−7^	
Maleic acid	649	8.59	1.87	0.011	1.23	
Oxalic acid	541	7.28	1.53	0.016	0.91	
Phenyl beta-d-glucopyranoside	480	14.01	1.75	0.030	1.32	Yes
Abietic acid	467	14.37	3.89	<0.001	0.09	
Itaconic acid	446	8.88	1.26	0.021	0.80	
Pyrophosphate	403	10.74	1.22	0.024	0.83	
Galactonic acid	396	12.58	1.47	0.031	0.80	Yes
Lyxose	384	10.66	1.31	<0.001	1.43	Yes
Lyxonic acid, 1,4-lactone	346	11.27	2.34	0.001	1.38	
5-Aminovaleric acid	322	10.61	1.38	<0.001	0.72	
*O*-methylthreonine	310	7.67	1.19	<0.001	1.50	Yes
Acetol	263	11.13	1.10	0.021	0.75	
3-Ureidopropionate	238	10.71	1.44	0.009	0.79	

**VEF *versus* NEF**						
Beta-mannosylglycerate	965	11.80	1.23	0.032	1.15	Yes
Tryptophan	890	13.66	2.90	<0.001	0.33	
2-Hydroxypyridine	865	6.59	1.13	0.047	1.19	Yes
Urea	793	8.19	2.38	<0.001	3.80	
Uric acid	745	13.00	3.59	<0.001	0.07	
Asparagine	713	10.84	1.31	0.010	1.23	Yes
Arachidic acid	681	14.55	3.06	<0.001	0.11	
Sucrose	656	15.30	3.66	<0.001	6.15 × 10^−7^	
Maleic acid	649	8.59	1.29	0.016	1.22	
Monostearin	545	16.04	2.26	0.033	2.43	Yes
Oxalic acid	541	7.28	1.25	0.016	0.90	
Uridine	469	14.65	1.13	<0.001	1.71	Yes
Abietic acid	467	14.37	3.57	<0.001	1.44 × 10^−7^	
Itaconic acid	446	8.88	1.52	0.003	0.78	
Pyrophosphate	403	10.74	1.50	0.004	0.83	
Valine	349	7.05	1.18	0.047	0.81	Yes
Lyxonic acid, 1,4-lactone	346	11.27	2.31	<0.001	1.56	
5-Aminovaleric acid	322	10.61	1.62	0.002	0.86	
Fructose	299	11.95	2.48	<0.001	0.14	
Thymol	283	8.69	1.27	0.012	0.91	Yes
4-Hydroxybutyrate	242	8.12	1.34	0.030	1.08	Yes
3-Ureidopropionate	238	10.71	1.80	<0.001	0.72	

**CVD *versus* NEF**						
Tryptophan	890	13.66	3.47	<0.001	2.47 × 10^−9^	
Urea	793	8.19	3.02	<0.001	6.81	
Uric acid	745	13.00	3.30	<0.001	8.82 × 10^−9^	
Arachidic acid	681	14.55	1.15	0.011	0.46	
Sucrose	656	15.30	3.23	<0.001	6.34 × 10^−7^	
Lactic acid	587	6.79	1.44	0.033	0.71	Yes
3-Aminoisobutyric acid	539	9.60	1.18	0.049	0.74	
2-Monopalmitin	487	14.95	2.43	0.005	0.15	Yes
Abietic acid	467	14.37	3.13	<0.001	1.48 × 10^−7^	
Thymidine	455	10.65	1.96	<0.001	0.13	
Oxalacetic acid	335	9.57	1.39	0.026	0.49	Yes
Glutamic acid	315	10.59	1.27	<0.001	0.23	
Fructose	299	11.95	2.20	<0.001	0.10	

a Fold change with a value >1 indicates a higher level in each comparison group. The *P* value was calculated using Student's *t* test. VIP, variable importance in the projection.

### Altered metabolic pathways related to different endothelial phenotypes

**Fig. 4:**
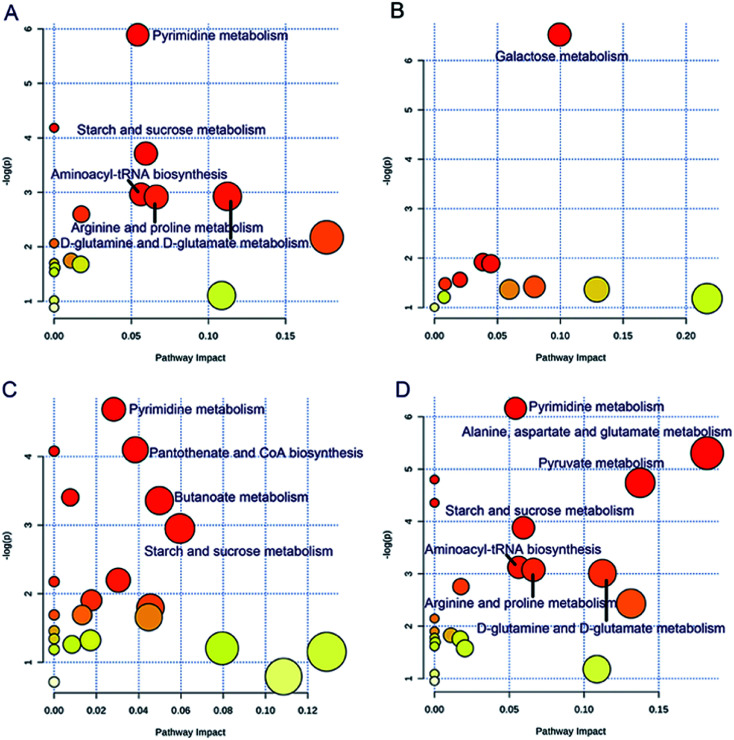
The disturbed metabolic pathways showing various metabolic changes when comparing (A) PEF *versus* NEF, (B) REF *versus* NEF, (C) VEF *versus* NEF, and (D) CVD *versus* NEF. NEF, normal endothelial function; PEF, cardiovascular risk-promoting endothelial function; REF, cardiovascular risk-resistant endothelial function; VEF, vulnerable endothelial function; CVD, cardiovascular disease.

The significantly altered metabolic pathways were revealed for the different endothelial phenotypes (Fig. 4). The same metabolic pathways were altered in the PEF and CVD groups, including pyrimidine metabolism, starch and sucrose metabolism, aminoacyl-tRNA biosynthesis, arginine and proline metabolism, and d-glutamine and d-glutamate metabolism. Additionally, alanine, aspartate and glutamate metabolism, and pyruvate metabolism were also perturbed in the patients with CVD. Meanwhile, galactose metabolism was exclusively disturbed in the REF group, while 4 metabolic pathways were clearly altered in the VEF group, including 2 unique pathways, namely, pantothenate and CoA biosynthesis, and butanoate metabolism.

The differential metabolites involved in the significantly altered metabolic pathways are shown in ESI Table 3.† For different comparisons, the ROC analyses showed that the area under the curve (AUC) values of the differential metabolites ranged from 0.734 to 1.000 in various metabolic pathways. Moreover, the AUC values were not significantly different after further adjustment for age and sex in the models (*P* values > 0.05).

## Discussion

In this metabolomics analysis of serum samples of 103 asymptomatic subjects and 12 CVD patients, some metabolites were altered across all of the different endothelial phenotypes, while other metabolites were exclusive to specific endothelial phenotypes, such as monoolein and 1-monopalmitin for the PEF phenotype, tagatose and lyxose for the REF phenotype, and valine and 4-hydroxybutyrate for the VEF phenotype. Furthermore, the altered metabolites were enriched in differential metabolic pathways, suggesting distinct metabolic patterns in response to a specific endothelial function status.

Impaired endothelial function was associated with higher cardiovascular risk in asymptomatic adults,^20,21^ which was mechanistically involved *via* pathological changes such as decreased NO synthesis, increased oxidative stress, and increased inflammation.^22^ Urea and glutamic acid, in arginine and proline metabolism, are connected to the ornithine cycle and the deamination of glutamic acid. Glutamic acid fluxes into the ornithine cycle as citrulline *via* deamination and subsequently synthesizes arginine in the cytoplasm;^23,24^ arginine is then converted to urea, which contributes to the excretion of excess ammonia. Not just an intermediate for ammonia excretion, arginine can also function as a precursor and immediately produce NO under the catalysis of endothelial NO synthase (eNOS). In this study, elevated urea and deceased glutamic acid may indicate active deamination in the form of urea and a relative reduction of NO synthesis in the subjects with PEF. Interestingly, elevation of urea has been proven to cause endothelial dysfunction *via* inducing reactive oxygen species (ROS) production in uremic mice.^25^ Uric acid and 3-aminoisobutyric acid were downregulated in the PEF group compared with the NEF group, suggesting increased oxidative stress according to *in vitro* and *in vivo* evidence.^26,27^ Additionally, tryptophan, an indicative essential amino acid for protein synthesis, was remarkably deficient in the PEF group. An inverse association between circulating tryptophan and inflammation has been confirmed in CVD patients by epidemiological studies.^28,29^

Active catabolism of pyrimidines was suggested in the PEF group, as shown by upregulated urea and downregulated 3-aminoisobutyric acid and thymidine, since urea is one of the main catabolic products of pyrimidine metabolism.^30^ As a terminal oxidation product of purines, a remarkable decrease in uric acid suggested a reduction of purine synthesis in the PEF groups. The noteworthy metabolite reductions in the purine and pyrimidine pathways have been confirmed in atherosclerotic plaques.^31^ Compared with the NEF group, the PEF group had invalid biosynthesis of aminoacyl-tRNA, which is crucial for growing polypeptide chains,^32^ suggesting that protein synthesis may be somewhat impaired in the PEF group. Additionally, as precursors of glucose, both sucrose and fructose were at low levels in the PEF, indicating an insufficient energy state. Notably, monoolein and 1-monopalmitin, both monoacylglycerol species of triglycerides, were somewhat elevated in the PEF group; similar circulating triglyceride species, instead of total triglycerides, have been proposed to increase cardiovascular risk in a population-based study.^33^ Briefly, the metabolic profiling of the PEF group was in line with the pathological mechanism of endothelial dysfunction, but also depicted an aberrant pattern in genomic stability, protein synthesis, energy supply, and fatty acid mechanism.

The change of carbohydrate metabolites and derivatives was noticeable in the subjects with REF, especially in tagatose, a monosaccharide sweetener. In contrast to sucrose, tagatose has a very low glycemic index and has a protective effect on lipid profiles, glycemia, and atherosclerosis *via* inhibition of hepatic glycogenolysis, intestinal glucose absorption, or excess adiposity.^34,35^ In this study, the relative increase of low-calorie sugar in galactose metabolism may contribute to the protection of endothelial function even under high cardiovascular risk. In addition, arachidonic acid, a polyunsaturated fatty acid, was specifically elevated for the subjects with REF. Evidence (*in vitro* and *in vivo*) has shown that arachidonic acid can promote NO release *via* evoking Ca^2+^ signals^36^ and exert an anti-inflammatory effect in the form of nitration,^37^ while impaired arachidonic acid metabolism can improve endothelial dysfunction.^38^ Therefore, the elevation of arachidonic acid may also be involved in the cardiovascular risk-resistant mechanism for the subjects with REF in this study.

For the subjects with VEF, the underlying mechanism remains poorly studied, although previous studies have suggested that unknown biological factors may contribute to atherosclerosis development even under a low-risk profile.^1,39^ Metabolite alterations of note, including the reduction of valine and 3-ureidopropionate and the elevation of maleic acid and 4-hydroxybutyrate, were involved in pantothenate and CoA biosynthesis (which are pivotal for CoA biosynthesis) and butanoate metabolism (which is pivotal for acetyl-CoA biosynthesis),^40,41^ thus supporting the relatively increased ratio of acetyl-CoA to CoA observed in the subjects with VEF. As the precursor of endogenous cholesterol biosynthesis, the abundance of acetyl-CoA is pertinent to not only cholesterol production but also eNOS function and oxidative stress through specific signaling pathways in vascular endothelial cells.^42,43^ Therefore, the dynamic imbalance between acetyl-CoA and CoA, characterized by relatively elevated acetyl-CoA, may contribute to endothelial dysfunction. In addition, although pyrimidine metabolism was disturbed in both the subjects with VEF and the subjects with PEF, in contrast with the depressed DNA metabolism observed in the subjects with PEF, perturbed RNA metabolism may exist in the VEF phenotype, as indicated by altered uridine and 3-ureidopropionate. Given the difference in genomic instability, further research is needed to determine the potential implications for the VEF phenotype.

CVD, the outcome of pathological development of vascular endothelial dysfunction, has been shown to have metabolic perturbations, such as decreased glycolysis and increased fatty acid oxidation in stable carotid plaques,^12^ a decrease in the tricarboxylic acid (TCA) cycle and phospholipid catabolism, higher branched-chain amino acid (BCAA) metabolites, and lower urea cycle metabolites in plasma samples.^44,45^ In this study, compared with the NEF group, lactic acid, oxalacetic acid and glutamic acid were substantially reduced in subjects with CVD. Lactic acid, a reduced product of pyruvate metabolism, is lower following decreased glycolytic flux;^12^ as a critical intermediate of the TCA cycle, oxalacetic acid can be derived from pyruvate *via* carboxylation, as well as from glutamic acid *via* glutamine oxidation, and plays a pivotal role in TCA cycle flux for tissue energy requirements.^46,47^ Therefore, these depressed metabolites may reflect the impaired energy metabolism and biosynthesis linked to the TCA cycle in CVD patients. Notably, we found that the metabolic profiles in the CVD patients, given the disturbed metabolic pathways, were mostly similar to the PEF group instead of the REF group or the VEF group, indicating the rationality of metabolic profiling in subjects with endothelial dysfunction.

Taken together, the metabolic profiles in the vascular endothelial dysfunction phenotype associated with high cardiovascular risk appear to mainly correlate with specific pathological changes. Meanwhile, disturbed galactose metabolism or an increased ratio of acetyl-CoA to CoA may contribute to the risk-resistant or vulnerable mechanism for vascular endothelial function. More importantly, for the primary or secondary care of cardiovascular disease, specific metabolite pools could be applied as potential circulating biomarkers to identify the individuals who may benefit from aggressive treatments despite low risk and those who should be provided less aggressive therapy despite high risk profiles.

Our study has several limitations. First, our subjects were not randomly sampled, and our findings should be replicated in other larger populations. Second, although GC-TOF-MS has good separation efficiency and detection sensitivity, it was unable to detect all of the metabolites; thus, its detection of metabolites may be biased. Finally, bioinformation about some metabolites, such as abietic acid, phenyl beta-d-glucopyranoside, and *O*-methylthreonine, is still unavailable. Therefore, our findings should be carefully interpreted.

## Conclusion

By characterizing the circulating metabolites in asymptomatic individuals and CVD patients, the current results clearly presented a diversity in metabolic patterns across different endothelial function phenotypes. From a metabolic standpoint, our findings may add to an understanding of the heterogeneous pathogenesis of early CVD in asymptomatic individuals with extreme cardiovascular risk.

## Author contributions

Q. Z. conceived and designed the experiments; Q. Z., B. M. and Y. Y. wrote the manuscript; B. M., Y. Y., Q. M. and C. Y. performed the experiments; Q. Z., B. M. and Y. Y. analyzed the data. All authors have read and approved the final manuscript.

## Conflicts of interest

There are no conflicts of interest to declare.

## Supplementary Material

RA-009-C9RA05526F-s001
