# Supplementary material for: Metabolic profiling reveals the heterogeneity of vascular endothelial function phenotypes in individuals at extreme cardiovascular risk
Source: RSC Adv. 2019 Sep 23;9(52):30033–44. doi: 10.1039/c9ra05526f (PMC9072126; doi:10.1039/c9ra05526f)
Supplement: RA-009-C9RA05526F-s001 [file RA-009-C9RA05526F-s001.pdf]

Electronic Supplementary Material (ESI) for RSC Advances.
This journal is © The Royal Society of Chemistry 2019

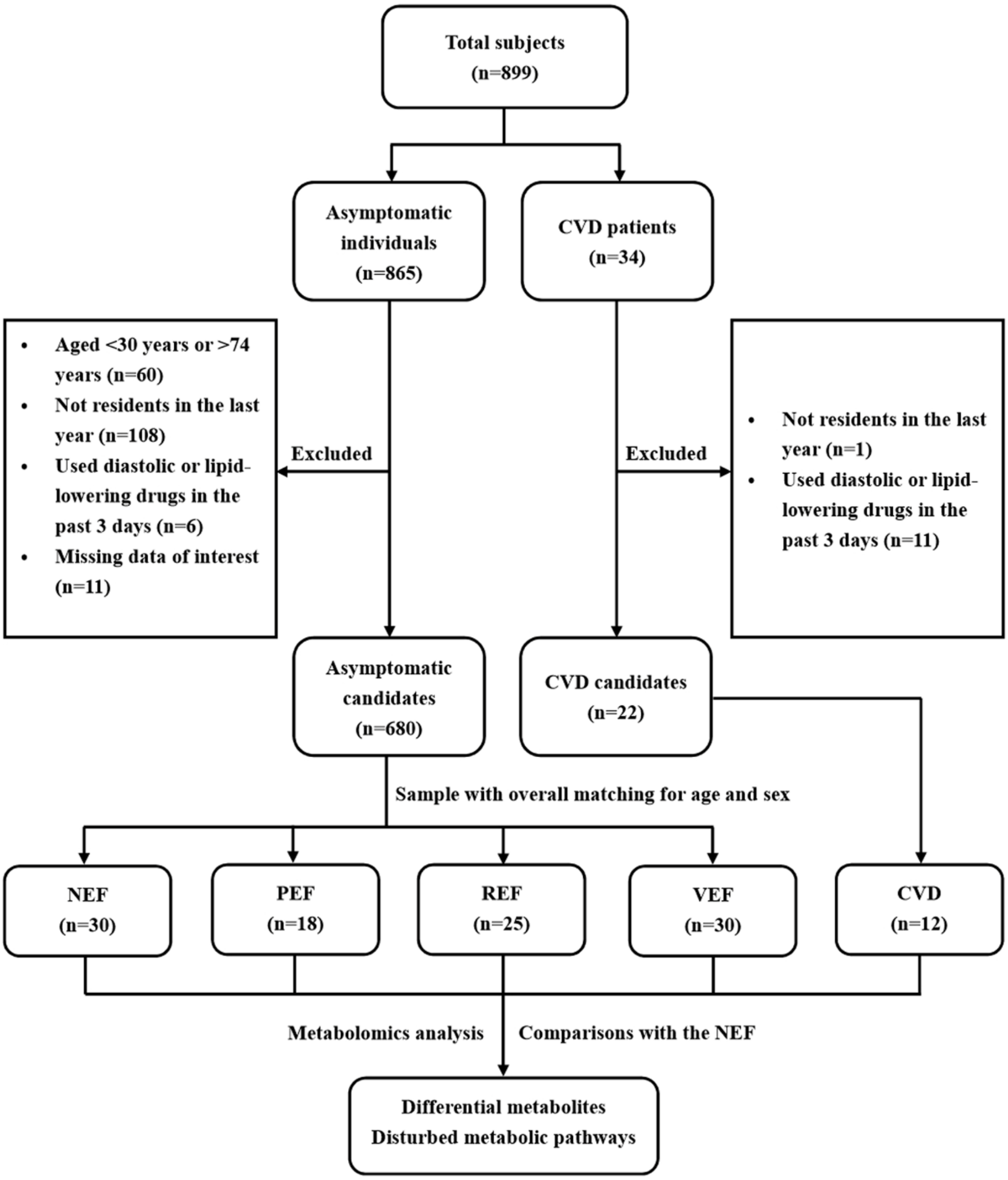

ESI Fig. 1. The selection criteria for the study population and flow diagram of the selection process. NEF, normal endothelial function; PEF, cardiovascular risk-promoting endothelial function; REF, cardiovascular risk-resistant endothelial function; VEF, vulnerable endothelial function; CVD, cardiovascular disease.

ESI Table 1. Characteristics of the study participants by different endothelial phenotypes

	NEF (n=30)	PEF (n=18)	REF (n=25)	VEF (n=30)	CVD (n=12)	*P* Value [d]
Age, y	55.3 (1.1) [a]	58.4 (1.9)	59.4 (1.3)	55.9 (1.1)	63.1 (2.8)	0.02
Male, %	40.0 (0.09) [b]	66.7 (0.11)	64.0 (0.10)	36.7 (0.09)	33.3 (0.14)	0.08
High school education, %	16.7 (0.07)	16.7 (0.09)	16.0 (0.07)	20.0 (0.07)	0	0.62
Race/ethnicity, %						0.68
Han	26.7 (0.08)	33.3 (0.11)	20.0 (0.08)	16.7 (0.07)	25.0 (0.12)	
Zhuang	53.3 (0.09)	33.3 (0.11)	56.0 (0.10)	66.7 (0.09)	58.3 (0.14)	
Other	20.0 (0.07)	33.4 (0.11)	24.0 (0.09)	16.6 (0.07)	16.7 (0.11)	
Current smoking, %	10.0 (0.05)	55.6 (0.12)	52.0 (0.10)	6.7 (0.05)	8.3 (0.08)	<0.001
BMI, kg/m^2	22.0 (0.5)	24.3 (1.1)	23.4 (0.6)	21.9 (0.5)	24.0 (1.1)	0.03
Systolic blood pressure, mmHg	121.4 (2.6)	158.5 (6.4)	150.6 (4.9)	120.2 (2.4)	134.9 (5.3)	<0.001
Heart rate, beat per minute	70.1 (1.5)	70.2 (2.6)	75.5 (2.8)	65.9 (1.6)	73.9 (2.8)	0.04
Serum CRP, mg/L	7.9 (6.4, 9.7) [c]	10.4 (5.5, 20.0)	9.4 (5.9, 14.9)	7.6 (6.5, 9.0)	11.7 (8.0, 16.9)	0.02
Total cholesterol, mg/dL	185.4 (6.8)	219.9 (8.7)	285.3 (28.1)	183.7 (5.0)	210.3 (13.4)	<0.001
Triglyceride, mg/dL	89.8 (73.3, 109.9)	208.9 (140.3, 311.1)	176.6 (121.8, 256.2)	81.2 (66.3, 99.6)	185.0 (128.2, 267.1)	<0.001
LDL cholesterol, mg/dL	97.4 (4.9)	117.4 (6.5)	156.8 (19.8)	97.3 (3.9)	110.2 (9.5)	<0.001
HDL cholesterol, mg/dL	57.1 (2.2)	44.5 (3.1)	53.2 (2.7)	58.5 (2.3)	50.1 (4.3)	0.01
Diabetes, %	6.7 (0.05)	16.7 (0.09)	16.0 (0.07)	0	25.0 (0.12)	0.04
FRS, % risk/10 years	6.0 (5.4, 6.6)	29.8 (25.5, 34.8)	29.0 (24.9, 33.9)	5.4 (4.7, 6.3)	NA	<0.001
FMD, %	12.8 (11.9, 13.8)	4.4 (3.6, 5.3)	11.8 (10.8, 12.9)	4.0 (3.4, 4.7)	7.8 (5.4, 11.2)	<0.001

[a] Mean (standard error) for continuous variables with normal distribution.

[b] Percent (standard error) for categorical variables.

[c] Geometric mean (95% CI) for continuous variables with nonnormal distribution.

[d] *P* value is calculated using the Wilcoxon signed-rank test for the continuous variable, using χ^2 analysis or Fisher's exact test when the variable is categorical.

BMI, body mass index; CRP, C-reactive protein; LDL, low-density lipoprotein; HDL, high-density lipoprotein; FRS, Framingham risk score; FMD, flow-mediated

vasodilation; NEF, normal endothelial function; PEF, cardiovascular risk-promoting endothelial function; REF, cardiovascular risk-resistant endothelial function; VEF, vulnerable endothelial function; CVD, cardiovascular disease.

ESI Table 2. The parameters for the OPLS-DA modeling quality by comparison groups

Comparison groups	N	R^2X (cum)	R^2Y (cum)	Q^2 (cum)
PEF versus NEF	48	0.133	0.983	0.892
REF versus NEF	55	0.124	0.925	0.697
VEF versus NEF	60	0.131	0.965	0.835
CVD versus NEF	42	0.14	0.983	0.866

OPLS-DA, orthogonal projections to latent structures discriminant analysis.

ESI Table 3. ROC analysis of altered metabolic pathways

Pathway	Metabolites (Fold Change) [a]	Combination	AUC (95% CI)	Sensitivity (%)	Specificity (%)	*P* Value [b]
PEF versus NEF						
Pyrimidine metabolism	urea (↑)<br>3-aminoisobutyric acid (↓)<br>thymidine (↓)	metabolites	1.000 (1.000, 1.000)	100.0	100.0	1.00
		metabolites + age + sex	1.000 (1.000, 1.000)	100.0	100.0	
Starch and sucrose metabolism	sucrose (↓)<br>fructose (↓)	metabolites	0.998 (0.993, 1.000)	100.0	96.7	0.48
		metabolites + age + sex	1.000 (1.000, 1.000)	100.0	100.0	
Aminoacyl-tRNA biosynthesis	tryptophan (↓)<br>glutamic acid (↓)	metabolites	0.989 (0.966, 1.000)	100.0	96.7	0.34
		metabolites + age + sex	1.000 (1.000, 1.000)	100.0	100.0	
Arginine and proline metabolism	urea (↑)<br>glutamic acid (↓)	metabolites	0.982 (0.944, 1.000)	100.0	96.7	0.37
		metabolites + age + sex	0.991 (0.971, 1.000)	100.0	96.7	
D-glutamine and D-glutamate metabolism	glutamic acid (↓)	metabolites	0.758 (0.608, 0.909)	66.7	83.7	0.09
		metabolites + age + sex	0.870 (0.758, 0.983)	77.8	86.7	
REF versus NEF						
Galactose metabolism	tagatose (↑)<br>sucrose (↓)<br>galactonic acid (↓)	metabolites	0.999 (0.995, 1.000)	100.0	96.7	0.48
		metabolites + age + sex	1.000 (1.000, 1.000)	100.0	100.0	
VEF versus NEF						
Pyrimidine metabolism	urea (↑)<br>uridine (↑)<br>3-ureidopropionate (↓)	metabolites	0.929 (0.865, 0.993)	96.7	76.7	1.00
		metabolites + age + sex	0.929 (0.866, 0.992)	96.7	76.7	

Pantothenate and CoA biosynthesis	valine (↓)<br>3-ureidopropionate (↓)	metabolites	0.779 (0.654, 0.903)	80.0	73.3	1.00
		metabolites + age + sex	0.779 (0.655, 0.903)	86.7	66.7	
Butanoate metabolism	maleic acid (↑)<br>4-hydroxybutyrate (↑)	metabolites	0.734 (0.603, 0.866)	80.0	70.0	0.37
		metabolites + age + sex	0.747 (0.616, 0.877)	80.0	73.3	
Starch and sucrose metabolism	sucrose (↓)<br>fructose (↓)	metabolites	0.996 (0.987, 1.000)	96.7	96.7	0.34
		metabolites + age + sex	0.999 (0.996, 1.000)	100.0	96.7	
CVD versus NEF						
Pyrimidine metabolism	urea (↑)<br>3-aminoisobutyric acid (↓)<br>thymidine (↓)	metabolites	0.994 (0.981, 1.000)	100.0	93.3	0.41
		metabolites + age + sex	1.000 (1.000, 1.000)	100.0	100.0	
Alanine, aspartate and glutamate metabolism	oxalacetic acid (↓)<br>glutamic acid (↓)	metabolites	0.850 (0.697, 1.000)	83.3	86.7	0.36
		metabolites + age + sex	0.906 (0.809, 1.000)	83.3	86.7	
Pyruvate metabolism	lactic acid (↓)<br>oxalacetic acid (↓)	metabolites	0.800 (0.665, 0.935)	91.7	63.3	0.42
		metabolites + age + sex	0.844 (0.727, 0.962)	100.0	63.3	
Starch and sucrose metabolism	sucrose (↓)<br>fructose (↓)	metabolites	1.000 (1.000, 1.000)	100.0	100.0	1.00
		metabolites + age + sex	1.000 (1.000, 1.000)	100.0	100.0	
Aminoacyl-tRNA biosynthesis	tryptophan (↓)<br>glutamic acid (↓)	metabolites	0.994 (0.981, 1.000)	100.0	96.7	0.48
		metabolites + age + sex	0.997 (0.990, 1.000)	100.0	96.7	
	urea (↑)	metabolites	0.989 (0.965, 1.000)	100.0	96.7	0.38

Arginine and proline metabolism	glutamic acid (↓)	metabolites + age + sex	0.997 (0.990, 1.000)	100.0	96.7	
D-glutamine and D-glutamate metabolism	glutamic acid (↓)	metabolites	0.810 (0.637, 0.983)	83.3	86.7	0.19
		metabolites + age + sex	0.897 (0.797, 0.997)	83.3	86.7	

[a] (↑) represents fold change >1, while (↓) represents fold change <1.

[b] *P* value is calculated using the z test.

ROC, receiver operating characteristic; AUC, area under the curve.
